# 

**Published:** 1896-12

**Authors:** 


### MARRIAGES.

At Montreal, Canada, September 23d, Dr. Cecil French, of Washington, D. C., was united in marriage to Miss Florence Day, second daughter of the late Mr. Edwin T. Day, barrister, of the city of Montreal, Canada. A trip, including many of the Eastern cities, followed the wedding breakfast and their return to Washington, Dr. French's home, where they expect to at once take possession of their new residence in the suburbs of the Capital city.

Harri H. Dell, of Sullivan, Illinois, graduate of McGill University, Class of '96, and former Secretary of the Montreal Veterinary Medical Association, was married September 30th to Miss L. E. Estelle Ratliffe, of Farmer City, Illinois.

October 14, 1896, at Broomall, Delaware County, Pa., by Rev. J. M. Keller, rector of St. David's Church, Dr. W. L. Rhoads, of Lansdowne, Pa., to Miss Anna M. Moore, of Broomall.

On October 27, 1896, at Haverhill, Mass., at the Trinity Episcopal Church, by the Rev. D. J. Ayers, Dr. John M. Parker, President of the Massachusetts State Veterinary Medical Association, and a member of the State Board of Cattle Commissioners, was married to Miss Edith Helen Snell. After the ceremonies the couple were driven to their new home, where a wedding lunch was served and a reception held.

Secretary.

### PENNSYLVANIA STATE BOARD OF VETERINARY MEDICAL EXAMINERS.

At the examination held at Harrisburg, October 20th and 21st, the following applicants successfully passed the Board: Drs. W. H. Yingst, Harrisburg, Pa., F. H. Schneider, Ashland, Pa., graduates of the New York College of Veterinary Surgeons, and Dr. J. H. Burt, of Union City, Pa. graduate of the Ontario Veterinary College.

The following members of the Board were present to conduct the examinations: President W. Horace Hoskins, Secretary S. J. J. Harger, and members J. W. Sallade and J. C. McNeil; absent, Dr. H. Walters.

The next examination of the Board will be conducted on December 20th and 21st, and will be specially for those who failed to pass the examination in June. The Board gives notice that during 1897 but two examinations will be held, the first in June and a second some six months later, in compliance with the requirements of the law, thus allowing those who failed to come up again for re-examination at the expiration of six months.

S. J. J. Harger,
Secretary.

W. Horace Hoskins,
President.

### PERSONAL.

### INDEX.

Abbott, A. V., 529
Abortion, result of, upon the milk, 292
Abscess of Steno's duct, aspiration-pneumonia following, 296
Abstract of a paper on Veterinary Dentistry, 720
of Stenographer's Report, U.S.V.M.A., 1895, 67, 232
Accidents observed on horses of pure English stock, 537
Actinomycosis, 116, 276
of the inferior maxilla, 726, 782
treatment of, 452, 471
Acute founder, arecoline, a new remedy against, 544
gastro-intestinal catarrh in the human body, parenchymatous mastitis as cause of, 292
Address of President Stewart before the Missouri Valley Veterinary Medical Association, 587
of Prof. Law at the inauguration of the New York State Veterinary College, 777, 805
of retiring President Harbaugh of Virginia State Veterinary Medical Association, 659
to the Association of Veterinary Faculties of N. A., 23
to the Students of Harvard Veterinary School, 753
Aftermath, 686, 738
After Buffalo—where? 684
Alabama, control work in, 414, 732
American canned meats, examination of, 787
horses for the French army, 201
Veterinary College, 435, 632
commencement exercises, 348, 488
resolutions adopted by Alumni Association, 571
Among the Colleges, 55
American Veterinary College, 435, 632
Chicago Veterinary College, 55, 751
College of Agricultural and Mechanical Arts, R. I., 562
Kansas City Veterinary College, 434, 633
McGill University, Veterinary Department, 751
McKillip Veterinary College, 55, 562, 631
National Veterinary College, 434, 751
New York College of Veterinary Surgeons, 55, 709
New York State Board of Regents, 434
New York State Veterinary College, 631, 801
Ohio Veterinary College, 55
Ontario Veterinary College, 54, 708
United States College of Veterinary Surgeons, 54, 632
Veterinary Department, Detroit Medical College, 434, 708
Veterinary Department, Harvard University, 631, 708
Veterinary Department, Ohio State University, 750
Veterinary Department, University of California, 562, 709
Veterinary Department, University of Pennsylvania, 561
Veterinary Schools in Austria, 55
Anæsthetic, petroleum-benzine as an, 823
Antitoxin, case of tetanus cured with, 33
Antitoxins, horse as a producer of, 13
Anthracoid diseases, 129
Aorta, embolism of, 298
Aphthous fever, immunity to, 389
immunizing action of iodide of potassium in regard to, 388
in a fœtus, 600
Appointments, U.S.V.M.A., 750
Apoplexy, parturient, 813
Archibald, R. A., 174
Arecoline, 544, 678
Army legislation, 52, 321, 414
Ascaris megalocephalis in the hog, 478
Aspiration-pneumonia following abscess of Steno's duct, 296
Association of Veterinary Faculties of North America, address to, 23
Association of Veterinary Faculties of North America, Society Proceedings of, 640, 745
At your leisure, 250
Austria, cattle legislation in, 467
control work in, 791
empirics in, 467
reform of veterinary studies in, 55
Swine plague in, 286, 390
Australia, control work in, 557, 837

Babb, Albert, 70, 638
Barium chloride, applicability of, in the treatment of colic, 44
experience with, 218, 662
Benner, W. G., 423
Berne, Sixth International Veterinary Congress in, 38, 122
Biart, E. H., 274, 590
Bird, Robert H., 270
Bitch, suppurative metritis in, 294
Bone-softening, 262
Bovine species, purpura hemorrhagica in, 199
Bray, T. A. 461
Breath of a dog injurious to health, 295
Bridge, F. S., 568
Brown, John E., 67, 211
Buffalo, 618
resolutions, 691
wins for 1896, 47
Bulletins received, 790

Calculus, urethral, in a horse, 402
in a steer, 553
California, control work in, 151, 694, 733
State Veterinary Medical Association, 154
Veterinary Department, University of, 562
Campaign of education, 681
Campbell, Lawrence, 846
Canada, control work in, 415, 557, 699
Canadian diplomacy and our cattle exports, 482
Cancer, cure of, 675
Canine cases, 294
distemper, 769
rabies in India, 782
tuberculosis, case of, 396
Cary, C. A., 99
Cape Colony, South Africa, control work in, 733
Carbolic acid, observations in regard to the application of, 467

Carotid artery, ligation of, 220
Castration, cryptorchids and their, 509
Cat, leukæmia in a, 398
Cataract in the horse, 677
Cattanach, John J., 786
Cattle, Belgian law against tuberculosis in, 289
  legislation in Austria, 467
  tick, extermination of, 649
  treatment of acute primary peritonitis in, 288
  vomiting among, 676
Cheap education, 721
Chicago Veterinary College, 55, 750
    commencement exercises, 427
   Medical Society, 845
Chickens, tuberculosis in, 461
Chit-chat, 163
Chloride of barium, treatment of colic with, 544
Civil-service examination questions, Department Public Safety, Philadelphia, 426
Clark, W. G., 340
College chart for 1896, 227
  of Agricultural and Mechanical Arts, 562
Colorado, control work in, 150
  Veterinary Medical Association, 850
Colt, intestinal obstruction in, 550
Commencement exercises:
  American Veterinary College, 348, 488
  Chicago Veterinary College, 427
  Indiana Veterinary College, 347
  Kansas City Veterinary College, 348
  McGill University, Veterinary Department, 428
  McKillip Veterinary College, 348
  National Veterinary College, 489
  New York College of Veterinary Surgeons, 348, 433
  Ohio Veterinary College, 427
  Ontario Veterinary College, 54, 429
  United States College of Veterinary Surgeons, 434
  Veterinary Department, Detroit Medical College, 488
  Veterinary Department, Harvard University, 631
  Veterinary Department, University of Pennsylvania, 561
Committee on Army Legislation, U.S.V.M.A., 52
  Intelligence and Education, U.S.V.M.A., Report of Chairman of, 97
Comparative Psychology Society, McGill University, 73, 345, 416, 496
Compulsory meat inspection and cattle insurance in Saxony, 207
Conference of the slaughter-house directors and representatives of the cities with public cattle-yards in Berlin, 667
Connecticut, control work in, 144, 409, 486, 696, 730
  Veterinary Medical Association, 71, 570
Control work:
  Alabama, 414, 732
  Australia, 557, 837
  Austria, 791
  California, 151, 694, 733
  Canada, 415, 557, 699
  Cape Colony, South Africa, 733
  Colorado, 150
  Connecticut, 144, 409, 486, 696, 730
  Delaware, 327
  District of Columbia, 53
  Great Britain, 328, 630
  Illinois, 413, 628, 698, 838
  Indiana, 487, 698, 732, 837
  Iowa, 414, 732
  Jamaica, 837
  Kansas, 487, 629
  Louisiana, 150, 628, 791
  Massachusetts, 144, 225, 327, 409, 487, 628, 730, 838
Control work:
  Maine, 149, 327, 628
  Michigan, 413, 628, 698
  Minnesota, 414
  Missouri, 327, 486, 629, 699, 732
  National, 151, 224, 414, 487, 630, 790
  Nebraska, 487
  New Hampshire, 696
  New Jersey, 53, 151, 327, 488, 557
  New York, 54, 149, 323, 411, 627, 697, 730, 791, 838
  Ohio, 698
  Oklahoma, 699
  Pennsylvania, 150, 224, 324, 411, 485, 557, 627, 697, 731, 837
  Rhode Island, 409
  South Dakota, 414
  Texas, 487
  Vermont, 149, 328, 410, 696, 838
  Virginia, 150, 413, 486, 731
  Wyoming, 150
Correction, 619
Correspondence, 839
  National Zoological Park, 484
  Osteoporosis not millet disease, 403
  Pasteur Monument Fund, 839
  "Query," What we want to know, 616
  Request for important information, 305
  Tale of an unsound hoise, 615
  Vaccination, 222
  Veterinary legislation, 304
Costly experience, 788
Cow, ovarian dropsy in, 725
  rib-fistula in, 287
Cryptorchids and their castration, 509
Currie, J. M , 705
Curtice, Cooper, 577, 649

Danger of living in glass houses, 480
Dangers of National Associations, 734
Dead horse, how its carcass is utilized in the arts, 597
Death of Count Taaffe, 209
Deaths caused by malignant pustules, 824
Defeat Senate Bill 1552, 483, 833
Delaware, control work in, 327
Dell, Harri H., 100, 154, 238, 336
Department of Public Safety, Philadelphia, civil-service examination questions, 426
Device, ingenious and useful, 477
Diagnostic agent, is mallein a specific as a, 596
Diphtheria, spread of, by house cats, 37
Disease, milk as a factor in the causation of, 253, 370
  navicular (navicular arthritis), 655
  pathogenesis of, 583
Diseases, contagious, transferred to mankind, 600
  of the hock-joint, 104
Disinfection of infected wounds by means of septic materials, 825
Distemper, canine, 769
District of Columbia, control work in, 53
    legislation in, 320, 561
    Veterinary Medical Association, 160
Dog, filaria in the eye of, 291
  intestinal obstruction in: its treatment, and the operations of laparotomy, enterotomy, enterectomy, and enterorrhaphy, 1
    parasitism in, and its treatment, 441
  myelitis in, 401
  pyæmia in, 100
  statistics on the frequency of neoplasms in, 543
  tuberculosis in, 536
  variability in the length of tail in, 608
Dogs, result of recent remarkable experiments with young, 592
Drake, H. L., 655
Driftwood, 647
Dunbar, W. A., 792
Duncan, J. T., 383

EDITORIALS, 230, 231, 310, 311, 312, 313, 406, 407, 482, 621, 681, 734, 735, 736, 789, 790, 833
Adopt this measure in the Smoky City, 555
Advertising one's profession and proprietary remedies, 135
After Buffalo—Where? 684
An unfortunate incident, 137
Another in the fold, 407
Another unsuccessful effort, 307
Appointment of State Veterinarian for Pennsylvania, 50
Better remuneration for scientific skill recognized, 135
Buffalo, 618
wins for 1896, 47
Campaign of education, 681
Canadian diplomacy and our cattle exports, 482
College chart for 1896, 227
Correction, 619
Costly experience, 788
Danger of living in glass houses, 480
Dangers of National Associations, 734
Defeat Senate Bill 1552, 483, 833
Don't monkey with a buzz-saw, 683
Examination of American canned meats in Germany, 787
Important notice, 737
JOURNAL change, 47
Legislation in Iowa, 136
in 1896, 228
Lingering embers of the spoils system, 307
Massachusetts Cattle Commission, 735
McKillip's loss, 136
New York registry bill, 483
Now for the Queen City of the Lakes, 556
Our added years, 618
Our April number, 306
Our Buffalo resolutions, 835
Our colleges, 621
Profession's interests above the individual, 309
Rank for our army veterinarians, 48
Sanitary police measures must be adopted, 787
Significant progress, 619
Slower method the truer one, 554
To our readers, 49, 832
Triumphant Massachusetts, 555
U.S.V.M.A., 788
Veterinary journalism, 480
legislation in New York State, 308
What will the others do? 481
What will our answer be? 620
Welcome 1897, 831
Educational, 840
Eliot, H. W., 71, 571
Ellis, Robt. W., 163, 242, 337, 401, 425, 494, 567, 793, 850
Embolism of the aorta, 298
Empirics in Austria, 467
English army, pension rules for, 678
Equine disease in Montana, 270
rabies, case of, 214
Every-day happenings, 854
Excision of the mammary gland, 612
Experience, unpleasant, though interesting, 211
Extermination of the cattle-tick, 649
Extracts from foreign journals:
Actinomycosis of the lower jaw, 782
treated with iodcalium; two cases, 471
Acute primary peritonitis in cattle, 288
Admission of women to the study of veterinary medicine, 675
American horses for the French army, 201
Apothecaries' against the veterinarians' right to dispense drugs, 43
Applicability of barium chloride in the treatment of colic, 44
Aphthous fever in a fœtus, 600
Arecoline, 678
Extracts from foreign journals:
a new remedy against acute founder, 544
Atresia ani et recti, operative treatment of, 291
Bacteriological course for veterinarians in Baden, 470
Belgian law against tuberculosis in cattle, 289
Bohemian Veterinary Institution at Prague, 673
Canine rabies in India, 782
Cataract in the horse, 677
Cattle legislation in Austria, 467
Celebration of the Belgian Veterinary Federation in honor of Prof. Alph. Degive, State Director of the School of Veterinary Medicine, 476
Compulsory meat inspection and cattle insurance in Saxony, 207
Conference of the slaughter-house directors and representatives of the cities with public cattle-yards in Berlin, 667
Contagious diseases transferred to mankind, 600
Cancer, cure of, 675
treatment of, with cancer-serum, 826
Death of Count Taaffe, 209
Deaths caused by malignant pustules, 824
Disinfection of infected wounds by means of septic material, 825
Empirics in Austria, 467
Establishment of a veterinary school in upper Austria, 671
Examination of the rectum for diagnostic purposes, 127
Export of glandered horses, 388
Fern extract as a remedy for tapeworm, 291
Filaria in the eye of a dog, 290
Fistula of the matrix, 605
For the prevention of mistakes in the handling of medicines, 469
Horseshoes, 601
Indemnification of owners for loss of animals with blackleg, 674
Idiosyncrasy against morphine, 600
Immunity to aphthous fever, 389
Immunizing action of iodide of potassium in regard to aphthous fever (foot and mouth disease), 388
Increase in the number of military veterinary surgeons, 469
Inoculation of tuberculosis of the gallinæ in the mammifers, 389
Internal inflammation of the eye of a horse, 669
Laws for stamping out cattle tuberculosis in France, 468
Malaria in animals, 204
Malignant œdema, 203
Mallein a specific as a diagnostic agent, 599
Malleination and tuberculination in point of a judicial examination, 540
Means of removing rust from instruments, 824
Melanosis of the subglossian ganglions resembling a gland in glanders, 611
Microbe producing results similar to the ray fungus, 782
Number of public slaughter-houses in the kingdom of Prussia, 42
Observations in regard to the application of carbolic acid, 467
On the use of turf as bedding, 607
Operation upon a fistula of the rectum, 785
Ovarian dropsy in a cow, 725
Palpation of the lymphatic ganglions accessible to the touch as a clinical diagnostic of tuberculosis, 609
Parenchymatous mastitis causing disease in humans, 292

Extracts from foreign journals:
Pension rules for the veterinary department of the English army, 678
Periodical lameness in the horse, 472
Petroleum-benzine as an anæsthetic, 823
Prolapsus vaginæ in a filly, 601
Question of the introduction of compulsory meat inspection and the insurance of cattle by the State in Germany, 43
Regulation of the district government in regard to the consumption of meat and fat which sticks to green hides, 44
Result of abortion upon milk, 292
Result of Pasteur inoculations, 38
Rib-fistula in a cow, 287
Rupture of the uterus, 608
Rye-bread with an admixture of malt as food for horses, 676
Septicæmia hæmorrhagica, 547, 722
Serum for tetanus, 388
therapeutics of tetanus, 667
Sixth International Veterinary Congress in Berne, 38, 122
Some accidents observed on horses of pure English stock, 537
remarks on the effects of drugs, 825
Spread of diphtheria by house-cats, 37
Statistics on the frequency of neoplasms in the dog, 543
Sterilization of tuberculous meat, 289
Sterilizing hair used in manufacture to prevent splenic fever and anthracoid diseases, 129
Stone in the bladder of a she-ass, 602
Strangles in South Africa, 202
Swine plague in Austria, 286, 390, 395
Stupefying animals before slaughter, 470
Tetanus, 388
cure by a sudden shock to the nerves, 470
Therapeutics of serum against tetanus, 37
Treatment of cancer with cancer serum, 826
of colic with chloride of barium, 544
of shoulder-boils or cold abscess of the shoulder, 207
Tuberculosis at the International Veterinary Congress, 473
in the dog; thirteen new cases, 536
of monkey, 386
Typhoid infection from drinking milk not thoroughly boiled, 823
Tyrol petitions for a veterinary school, 675
Variability in the length of tail in the dog, 608
Vomiting among cattle, 676

FAVILLE, GEO. C., 240, 258, 375, 640
Far and near, 739
Felton, Howard B., 769
Fern extract as a remedy for tapeworm, 291
Ferster, J. H., 624
Filaria in the eye of a dog, 290
Filly, prolapsus of the vaginæ in, 601
Fistula of the matrix, 605
of the rectum, operation upon, 785
Fistulous withers treated by actual cautery, 728
Flea, lively, a wonderfully constructed and pugnacious little pest, 596
Fœtus, aphthous fever in, 600
Forbes, John, 281
Formalin as a preservative fluid, 383
For the prevention of mistakes in the handling of medicines, 469
Fracture of the radius, 215
of the trapezium, 302
Frame, D. P., 851
French, Cecil, 1, 294, 441, 484, 551, 822, 841
army, American horses for, 201
From many sources, 712
Fridirici, U. G., 570

GARDNER, J. H., 623
Gaunt, P. F., 71
Gelsemium, fluid extract of, in tetanus, 680
Gill, H. D., 746, 842
Glanders, melanosis of the subglossian ganglions resembling a gland in, 611
in horses, 274
Glass houses, danger of living in, 480
Gleanings, 137, 223, 285, 408, 415, 458, 466, 479, 505, 595, 642, 699, 710, 733, 743, 751, 804
Glennon, J. T., 462
Golden-rod killing horses, 132
Goldstein, Samuel, 459
Good, Joseph M., 301
Great Britain control work in, 328, 630
Greer, John, Jr., 296
Greiner, L A., 402
Gurley, R. R., 305

HAGENBURGER, G. LEO, 613
Harbaugh, W. H., 177, 640, 679
Hart, John R., 491, 568
Harger, S. J. J., 18, 249, 851
Health of the pig, 184
Helmer, Jacob, 759
Hereditary transmission of tuberculosis, 456
Heredity, 265, 378
Hernia, traumatic ventral, 201
Hickman, R. W., 364
Hinebauch, T. D., 333, 405
Hinman, W. J., 158
Hock-joint, diseases of, 104
Hog, ascaris megalocephalis in, 478
Hoge, M. D., 262
Hollingsworth, W. G., 680
Hopkins, F. W., 820
Homœopathy in veterinary practice, 529, 759
Horse as a producer of antitoxins, 13
-breeding, 522
cataract in, 677
dead, how its carcass is utilized in the arts, 597
internal inflammation of the eye, 669
periodical lameness in, 472
tale of an unsound, 615
undescended testicle in, 94
urethral calculus in, 402
Horses, American, for the French army, 201
glanders in, 274
golden-rod killing, 132
Hoskins, J. Preston, 467, 536, 599, 667, 722, 782, 823
W. Horace, 249, 563, 851
How shall the practitioner continue his education? 174
Hunter, S. L., 158, 495, 569
Hurlburt, R. C., 720
Hybridism, peculiar case of, 822

ILLINOIS, control-work in, 413, 628, 698, 838
State Veterinary Medical Association, 70, 638
Immunity, theories of, 189
to aphthous fever, 389
Immunizing action of iodide of potassium in regard to aphthous fever, 388
Important notice, 737
Increase in the number of military veterinary surgeons, 469
Indiana, control work in, 487, 698, 732, 837
Veterinary College, commencement exercises, 347
Inferior maxilla, actinomycosis of, 726
Influence of fever on a case of traumatic tetanus, 35
Ingenius and useful device, 477
Injury, peculiar, 786
Inoculation of tuberculosis of the gallinæ in the mammifers, 389
Instruments, means of removing rust from, 824
Interesting canine cases, 294

Internal inflammation of the eye of the horse, 669
International Veterinary Congress in Berne, 38, 122
tuberculosis at, 473
Intestinal obstruction in a colt, 550
parasitism in the dog, and its treatment, 441
Intussusception, 612
Iodcalium, two cases of actinomycosis treated with, 471
Iowa, control work in, 414, 732
legislation in, 136, 141
State Veterinary Medical Association, 58
Is mallein a specific as a diagnostic agent? 599

JAMAICA, control work in, 837
Jobson, George, 33
Johnson, G. A., 85
Jopling, William, 245
Journals, review of, 827

KANSAS CITY VETERINARY COLLEGE, 434, 633
commencement exercises, 348
control work in, 487, 629
Keystone Convention, echoes of, 343
Veterinary Medical Association, 71, 160, 246, 337, 490, 495, 567, 705, 843
Kingery, S. H., 27
Klutts, L. M., 215

LATHAM, JOHN, 116
Law, James, 777, 805
Laws, J. P., 73
Leach, G. Ed., 194
Legislation, Army, 52, 321, 414
in District of Columbia, 320, 561
Iowa, 136, 141
Manitoba, 322
Maryland, 51
National, 320, 560
New Jersey, 317
New York, 138, 315, 560
Ohio, 320
Oregon, 561
Pennsylvania, 319, 558
Lellman, Wilfried, 398
Leukæmia in a cat, 398
Ligation of the carotid artery, 220
Lingering embers of the spoils-system, 307
Lion, paralysis in, 551
Longevity of skye-terriers, 295
Louisiana, control work in, 150, 628, 791
Lungs, nodes found in, 462
Lusson, L. O., 299
Lyman, R. P., 550

MAINE, control work in, 149, 327, 628
Veterinary Medical Association, 240, 703
Malaria in animals, 204
Malignant œdema, 203
Malleination and tuberculination in point of judicial examination, 540
Mammary gland, excision of, 612
Manitoba, legislation in, 322
Veterinary Medical Association, 156, 420, 499, 792
Marriages among veterinarians, 50, 231, 408, 562, 836
Massachusetts Cattle Commission, 735
control work in, 144, 225, 327, 409, 487, 628, 730, 838
Veterinary Medical Association, 333, 421, 571, 838
Martin, W. J., 104, 302, 612
Matrix, fistula of, 605
Maryland, legislation in, 51
McCarrey, J. J., 218
McGill University, 471, 751
commencement exercises, 428
Comparative Psychology Society, 73, 345, 416, 496
McGill University, valedictory address to the graduates in veterinary science, 357
McIntosh, D., 184
McKillip Veterinary College, 55, 136, 562, 631
commencement exercises, 348
McNaught, David, 477
Means of removing rust from instruments, 824
Medicines, for the prevention of mistakes in the handling of, 469
Melanosis of the subglossian ganglions, 611
Merillat, E., 516
L. A., 617
Michener, J. Curtis, 813
Michigan, control work in, 413, 628, 698
State Board of Health, 491
Veterinary Medical Association, 73, 159, 242
Milk as a factor in the causation of disease, 253, 370
result of abortion upon, 292
Miller, Frank H., 388, 396
Mills, Wesley, 357
Minnesota, control work in, 414
Missouri, control work in, 327, 486, 629, 699, 732
State Veterinary Medical Association, 641
Valley Veterinary Medical Association, 158, 495, 568
Address of President Stewart before, 587
Molar, misplaced, 302
Monkey, tuberculosis of, 386
Montreal Veterinary Medical Association, 152, 236, 333, 426, 847
Moore, R. C., 509
Morris, Claude D., 641
E. H., 220
Morphine, idiosyncrasy against, 600
Myelitis in a dog; recovery, 401

NATIONAL control work, 151, 224, 414, 487, 630, 790
legislation, 320, 560
Veterinary College, 434, 751
commencement exercises, 489
Navicular disease (navicular arthritis), 655
Nebraska, control work in, 487
Veterinary Medical Association, 420
Neher, H., 223
Neoplasm in the dog, statistics on the frequency of, 543
Nerves, tetanus cured by a sudden shock to, 470
Nerve-suturing, 552
Ness, J. A., 522
Netherton, G. T., 199
New Hampshire, control work in, 696
Veterinary Medical Association, 245, 494, 705, 744
New inventions, 83, 168, 351, 507, 648, 687, 714
New Jersey, control work in, 53, 151, 327, 488, 557
legislation in, 317
New publications, reviews of:
Bulletin No. 12, U. S. Department of Agriculture, 666
Diagrams of the Horse. Prof. Sussdorf, M.D.; translated by W. Owen Williams, 625
Dog in Health and Disease. Prof. Wesley Mills, 51
Electricity in Electro-therapeutics. Edwin J. Houston, Ph. D., and A. E. Kennelly, Sc.D., 314
Fleming's Veterinary Obstetrics, 313
Gould's Students' Medical Dictionary, 10th edition, 626
How to Disinfect, 51
Illustrated Dictionary of Medicine, Biology, and Allied Science. George M. Gould, A.M., M.D., 314
Kobert's Practical Toxicology, 666
Outlines of Veterinary Anatomy. Prof. O. Chranock Bradley, 625

New publications, reviews of:
Practical Toxicology, 51
Report of Massachusetts Cattle Commission, 252
Translation of C. Pellerin's Median Neurotomy in the Treatment of Chronic Tendonitis and Periostitis of the Fetlock. A. Liautard, 625
Veterinary Compend of Materia Medica and Therapeutics. A. C. Hassloch, 626, 666
Veterinary Herald, 252
New York College Veterinary Surgeons, 55, 709
commencement exercises, 348, 433
New York, control work in, 54, 149, 323, 411, 627, 697, 730, 791, 838
legislation in, 138, 560
registry bill, 483
New York State Board of Regents, 434
Veterinary College, 631, 801
address of Prof. Law at the inauguration of, 777, 805
Examining Board, legislation of, 315
Medical Society, 419, 574, 641, 746, 798
Niagara and Orleans County Veterinary Medical Association, 641, 846
Niles, E. P., 189
W. B., 169
Noack, Otto, 72, 108, 298, 338, 388, 495, 744
Nodes found in the lungs, notes on, 462
Non-classified disease affecting range horses in Montana, 270
North Dakota Veterinary Medical Association, 330
Notes on veterinary education and intelligence, concerning veterinary and agricultural colleges, 169
Nutrition, 516

OBITUARY of Carlin, Nat., 231
Custer, W. U., 50
Douglas, Charles F., 737
Gadsden, John W., 685
Hamill, James, 622
Leich, George, 686
McLaughlin, W. T. J., 231
Navin, John N., 231
Rayner, Moncure R., 624
Roberge, David, 623
Rosekrans, Mrs. William R., 686
Southwick, W. D., 737
Smith, W. E., 624
Taaffe, Count, 209
Willits, Edwin, 836
Œdema, malignant, 203
Œsophageal obstruction, peculiar fatal result, 130
Of passing interest, 81
Ohio, control work in, 698
legislation in, 320
State Veterinary Medical Association, 248
Veterinary College, 55
commencement exercises, 427
Oklahoma, control work in, 699
Oneida County Veterinary Medical Association, 705
Ontario Veterinary College, 708
commencement exercises, 54, 429
Society proceedings, 73, 159, 340, 793
On the use of formalin as a preservative fluid, 383
turf as bedding, 607
Oregon, legislation in, 561
Osteoporosis, 258, 375
not millet disease, 403
Our added years, 618
April number, 306
Buffalo resolutions, 835
colleges, 621
Our profession, 820
Ovarian dropsy in a cow, 725

PARALYSIS in a lion, 551
Parke, Davis & Co., 508
Parker, John M., 253, 370
Parturient apoplexy, 813
Pasteur inoculations, result of, 38
Pathogenesis of disease, 583
Peculiar fatal result of an œsophageal obstruction, 130
injury, 786
Pennsylvania, appointment of State Veterinarian for, 50
control work in, 150, 224, 324, 411, 485, 557, 627, 697, 731, 837
legislation in, 319, 558
State Veterinary Medical Association, 250, 341, 422, 571, 642, 706, 794
Examining Board, 249, 425, 563, 851
Periodical lameness in the horse, 472
Peritonitis in cattle, treatment of acute primary, 288
Personal, 32, 36, 79, 166, 210, 353, 436, 484, 503, 575, 598, 644, 713, 741, 801, 851
Peters, A. T., 420
Petroleum-benzine as an anæsthetic, 823
Pig, health of, 184
Plaskett, Joseph, 662
Plea for the institution of a new special course of instruction in Veterinary Colleges, 459
Pleuro-pneumonia, preventive inoculation against, 590
sporadic, 464
Pope, L. Jr., 246, 495, 705
Prague, Bohemian Veterinary Institution at, 673
Price, R., 499
Prof. Horday on "New Instruments," 120
Prolapsus vaginæ in a filly, 601
Public slaughter-houses in the Kingdom of Prussia, number of, 42
Purpura hemorrhagica in the bovine species, with report of case, 199
Pyæmia in the dog, 100

"QUERY," what we want to know? 616

RABIES, equine, case of, 214
canine, in India, 782
Radius, fracture of, 215
Rectum, examination of, for diagnostic purposes, 127
Reform of veterinary studies in Austria, 55
Reports of cases:
Actinomycosis of the inferior maxilla in a horse, 726
Ascaris megalocephalis in the hog, 478
Aspiration-pneumonia following abscess of Steno's duct, 296
Canine tuberculosis, case of, 396
Cow with chronic cough that did not react after repeated injections of tuberculin, 299
Dislocation of the head of the femur, separation of the pubic femoral ligament from the femur, and rupture of the capsular ligament, 786
Embolism of the aorta, 298
Equine rabies, 214
Experiments with barium chloride, 218
Excision of the mammary gland, 612
Fistulous withers treated by actual cautery, 728
Fluid extract of gelsemium in tetanus, 680
Fracture of radius, 215
of the trapezium, 302
Golden-rod killing horses, 132
Hybridism, peculiar case of, 822
Influence of fever on a case of traumatic tetanus, 35
Ingenious and useful device, 477

Reports of cases:
Intestinal obstruction in a colt, 550
Intussusception, 612
Leukæmia in a cat, 398
Ligation of the carotid artery, 220
Misplaced molar, 302
Myelitis in a dog; recovery, 401
Nerve suturing, 552
Paralysis in a lion, 551
Peculiar case of hybridism, 822
fatal result of an œsophageal obstruction, 130
injury, 786
Some interesting canine cases, 294
Subnormal temperature, case of, 679
Tetanus cured with antitoxin, 33
Traumatic ventral hernia, 301
Unpleasant though interesting experience, 211
Urethral calculus in a horse, 402
steer, 553
Report of the Chairman of Committee on Intelligence and Education, U. S. V. M. A., 97
on Southern cattle fever, 177
of a case of subnormal temperature, 679
Request for important information, 305
Resolutions, Buffalo, 691
Results of abortion upon the milk, 292
of Pasteur inoculations, 38
Review of journals, 827
Rhoads, W. L., 248, 338, 491, 568, 583, 706, 845
Rhode Island, control work in, 409
Rib-fistula in a cow, 287
Ripples, 535, 549
Rogers, Howard P., 333, 422, 571
Rupture of the uterus, 608
Russell, W. T., 265, 378
Rutherford, 499
Ryder, H. R., 73
J. E., 79

Sallade, J. W., 717
Salmon, D. E., 839
Sanitary police measures must be adopted, 787
Saxony, compulsory meat inspection and cattle insurance in, 207
Schaefer, V., 452
Schuylkill Valley Veterinary Medical Association, 72, 250, 338, 495, 569, 744
Schwarzkopf, Olof, 13, 640
Scott, J. L., 132
Septicæmia hæmorrhagica, 547, 722
Serum-therapeutics, 18
of tetanus, 667
She-ass, stone in the bladder of, 602
Shoulder boils or cold abscess of the shoulder, treatment of, 207
Significant progress, 619
Sisson, S., 94
Sixth International Veterinary Congress in Berne, 38, 122
Smith, V. P., 553
Social position of the veterinarian, 717
Society proceedings:
Alumni Association, American Veterinary College, 571
Associations and association meetings, 748, 842
Association of Veterinary Faculties of North America, 640, 745
California State Veterinary Medical Association, 154
Chicago Veterinary Society, 845
Colorado State Veterinary Medical Association, 850
Comparative Psychology Society, McGill University, 73, 345, 416, 496
Connecticut Veterinary Medical Association, 71, 570
District of Columbia Veterinary Medical Association, 160

Society proceedings:
Illinois State Veterinary Medical Association, 70, 638
Iowa State Veterinary Medical Association, 58
Keystone Veterinary Medical Association, 71, 160, 246, 337, 490, 495, 567, 705, 843
Maine Veterinary Medical Association, 240, 703
Manitoba Veterinary Medical Association, 156, 420, 499, 792
Massachusetts Veterinary Medical Association, 333, 421, 571
Michigan State Board of Health, 491
Veterinary Medical Association, 73, 159, 242
Missouri State Veterinary Medical Association, 641
Valley Veterinary Medical Association 158, 495, 568
Montreal Veterinary Medical Association, 152, 236, 333, 426, 847
Nebraska Veterinary Medical Association, 420
New Hampshire Veterinary Medical Association, 245, 494, 705, 744
New York State Veterinary Medical Society, 419, 574, 641, 746, 798
Niagara and Orleans County Veterinary Medical Association, 641, 846
North Dakota Veterinary Medical Association, 330
Ohio State Veterinary Medical Association, 248
Oneida County Veterinary Medical Association, 705
Ontario Veterinary Association, 159, 340
Medical College Society, 73, 793
Pennsylvania State Veterinary Medical Association, 250, 341, 422, 571, 642, 706, 794
Examining Board, 249, 425, 563, 851
Schuylkill Valley Veterinary Medical Association, 72, 250, 338, 495, 569, 744
United States Veterinary Medical Association, 67, 232, 329, 418, 572, 635, 700, 750, Nos. 10, 11, 12, pages 1-96
Veterinary Medical Association of New York County, 78, 162, 241, 336, 423, 493, 566, 792, 849
Veterinary Medical Association of New Jersey, 493
Virginia State Veterinary Medical Association, 238, 639
Western Iowa Veterinary Medical Association, 70
Wisconsin Society of Veterinary Graduates, 73, 339
Wyoming Valley Veterinary Medical Association, 425
Some diseases of the hock-joint, 104
remarks bearing on tuberculosis, 577
Southern cattle fever, report on, 177
South Africa, strangles in, 202
Spencer, H. A., 156
Spoils-system, 307
Sporadic pleuro-pneumonia, 464
Spread of diphtheria by house cats, 37
Statistics of the frequency of neoplasms in the dog, 543
Steer, urethral calculus in, 553
Sterilization of tuberculous meat, 289
Sterilizing hair used in manufacture to prevent splenic fever, 129
Stone in the bladder of a she-ass, 602
Strangles in South Africa, 202
Stupefying animals before slaughter, 470
Subnormal temperature, report of a case, 679
Sugden, B. A., 847
Suppurative metritis in a bitch, 294
Sweeny, Thomas M., 640
Sweetapple, C. H., 159, 794
Swine plague in Austria, 286, 390

TALE of an unsound horse, 615
Tapeworm, fern extract as a remedy for, 291
Technique of the tuberculin test, 85
Tetanus, 27
case of, cured with antitoxin, 33
cured by a sudden shock to the nerves, 470
fluid extract of gelsemium in, 680
serum-therapeutics of, 667
therapeutics of serum against, 37
traumatic, the influence of fever on a case of, 35
Texas, control work in, 487
There is always something new, 348
The State and veterinary science, 364
Therapeutics of serum against tetanus, 37
Theories of immunity, 189
Thomson, J. P., 641, 847
Thomas, F. A., 464
Thurston, E. B., 130
Timberman, J. H., 425
To our readers, 49
Transactions Thirty-third Annual Meeting United States Veterinary Medical Association, Buffalo, N. Y., September, 1896, Nos. 10, 11, 12:
Address of President, 83
Committee on Army Legislation, Report of, 23
on Diseases, Report of, 55
on Intelligence and Education, 71
on Publication, Report of, 49
Gill, H. D., the relation of horseshoers to veterinarians and veterinary colleges, 90
Minutes, 1
President's address, 83
Relation of horseshoers to veterinarians and veterinary colleges, 90
Report of Committee on Army Legislation, 23
on Diseases, 55
on Intelligence and Education, 71
on Publication, 49
Secretary's Report, 29
State Secretaries' Reports, 31–33
Translations and suggestons, 613
Trapezium, fracture of, 302
Traumatic ventral hernia, 301
Treatment of actinomycosis, 452
cancer with cancer serum, 826
colic with chloride of barium, 544
shoulder-boils or cold abscess of the shoulder, 207
strychnine-poisoning by hypodermic injections of chloral hydrate, 296
Trichinosis, 108
Triumphant Massachusetts, 555
Tuberculin, repeated injections of, in a cow with chronic cough, 299
Tuberculosis, 281
at the International Veterinary Congress, 473
canine, 396
hereditary transmission of, 456
in cattle, Belgian law against, 289
in chickens, 461
in the dog, thirteen new cases of, 536
of monkeys, 386
remarks of Drs. Price and Rutherford on, 499
some remarks bearing upon, 577
Tuberculous meat, sterilization of, 289
Turf as bedding, on the use of, 607
Tuttle, L. E., 745
Typhoid infection from drinking milk not thoroughly boiled, 823
Tyrol petitions for a veterinary school, 675

UNDERHILL, B. M., 214
Undescended testicle in the horse, 94
Unfortunate incident, 137
Unpleasant though interesting experience, 211
Use of formalin as a preservative fluid, 383
United States College of Veterinary Surgeons, 54, 434, 632
commencement exercises, 434
Veterinary Medical Association, 52, 329, 418, 572, 635, 788
abstract of stenographer's report, 1895, 67, 232
appointments, 750
meeting-place for 1897, 734
proceedings, 700; Nos. 10, 11, 12, pages 1-96
Urethral calculus in a horse, 402
in a steer, 553
Uterus, rupture of, 608
Useful formulas, 841
Useful and ingenious device, 477

VETERINARIAN, social position of, 717
Vermont, control work in, 149, 328, 410, 696, 838
Veterinarians in Baden, bacteriological course for, 470
Veterinary colleges, a plea for the institution of a new special course of instruction in, 459
dentistry, abstract of a paper on, 720
Department, Detroit Medical College, 434 488, 708
Harvard University, 631, 708
Ohio State University, 750
University of California, 562, 709
University of Pennsylvania, 561
education and intelligence, concerning veterinary and agricultural colleges, notes on, 169
Faculties of North America, Association of, 640, 745
journalism, 480
legislation in New York State, 308
meat-inspection, 194
Medical Association of New Jersey, 493
of New York County, 78, 162, 241, 336, 423, 493, 566, 792, 849
medicine, admission of women to the study of, 675
practice, homœopathy in, 529,759
school in upper Austria, establishment of 671
science and the State, 364
Virginia, control work in, 150, 413, 486, 731
State Veterinary Medical Association, 238, 639
address of retiring President Harbaugh, 659
Vomiting among cattle, 666

WALKER, R. G, 305, 617
Ward, S. H., 456
Wattles, J. H., 23
Waugh, James A., 728
Webber, L. R., 612
Welcome, 1897, 831
Wende, John, 302
Western Iowa Veterinary Medical Association, 70
West, W. L., 241, 704
What we want to know, 508, 616, 634
What will our answer be? 620
What will the others do? 481
Wheeler, A. S., 35
Why we progress, 439
Whitney, William F., 753
Willyoung, L. E., 726
Wingate, J. L., 276
Wisconsin Society of Veterinary Graduates, 73, 339
Wyman, W. E. A., 552
Wyoming, control work in, 150
Valley Veterinary Medical Association, 425

Youngberg, A., 478

